# A new species of *Megastigmus* (Hymenoptera, Megastigmidae) from China

**DOI:** 10.3897/BDJ.11.e102828

**Published:** 2023-04-11

**Authors:** Xiaoxiao Chen, Jingge Kuang, Wenjing Tao, Zhongping Xiong, Kangshan Mao

**Affiliations:** 1 Key Laboratory for Bio-Resource and Eco-Environment of Ministry of Education & Sichuan Zoige Alpine Wetland Ecosystem National Observation and Research Station, College of Life Science, Sichuan University, Chengdu 610065, Sichuan, China Key Laboratory for Bio-Resource and Eco-Environment of Ministry of Education & Sichuan Zoige Alpine Wetland Ecosystem National Observation and Research Station, College of Life Science, Sichuan University Chengdu 610065, Sichuan China; 2 College of Forests, Southwest Forestry University, Kunming 650224,Yunnan, China College of Forests, Southwest Forestry University Kunming 650224,Yunnan China

**Keywords:** morphology, DNA barcoding, phylogenetic relationships, genetic distance

## Abstract

**Background:**

Most species of *Megastigmus* are considered important economic pests that grow in seeds, especially of conifers. Accurate identification of species is a crucial step for the biological research of parasitic pests and the further application of biological control. However, their large variety, small size, similar morphology and different growth and development stages have brought great challenges to taxonomic research. Traditional morphological identification often takes a long time and this requires us to seek a new method for rapid and accurate identification. Therefore, the better identification of *Megastigmus* urgently needs to be combined with molecular methods to help taxonomic development.

**New information:**

Here, *Megastigmusdaduheensis* sp. n. (Chalcidoidea: Megastigmidae) was identified, based on morphology and molecular markers, such as *COI* and *Cytb*. *M.daduheensis* sp. n. is distinct from other known species of the same genus in the morphology. The results of the molecular phylogenetic tree, similarity alignment and genetic distance indicate that the *COI* and *Cytb* sequences of *M.daduheensis* sp. n. are highly similar to *M.sobinae* and *M.duclouxiana*, but there are some genetic differences. The genetic distances of *M.daduheensis* sp. nov. with *M.duclouxiana* and *M.sabinae* were 0.34 and 0.33 and the percentages of shared base pairs were 76.3% and 76.8%, respectively. Both morphological and molecular data classified *M.daduheensis* sp. n. as a new species. The obtained *COI* and *Cytb* sequences of *M.daduheensis* sp. n. can be used as DNA barcodes, providing molecular data for rapid and accurate identification of this species in the future.

## Introduction

Megastigmidae comprises over 200 species currently classified into 12 valid genera which are generally considered to have a worldwide distribution and *Megastigmus* is the only cosmopolitan genus of the family (Böhmová et al. 2022). *Megastigmus* was described by Dalman (1820) as the subgenus of *Torymus* Dalman, with *Pteromalusbipunctatus* Swederus, 1795 as its type species. Later, *Megastigmus* was recorded as a valid genus by several scholars and Crosby (1913) designated its type species as *P.bipunctatus* (Curtis 1829, Dalla 1898, Ashmead 1904).

*Megastigmus* is a diverse group with 144 valid species described in the Universal Chalcidoidea Database (Noyes 2019). Of these, most species have been described from the Holarctic and Australasian Regions (Bouček 1988, Grissel 1999). So far, 18 species in *Megastigmus* have been reported from China. The species of *Megastigmus* display a great variety of feeding strategies in which many species are seed feeders and others are mostly solely carnivorous (Roques and Skrzypczynska 2003, Böhmová et al. 2022). Their habit leads to a decrease in seed yield which seriously endangers the reproduction of trees. We need to quickly identify and control the species to reduce damage. However, their small size and similar appearance makes it difficult to identify. The identification of closely-related species can no longer be met by using morphological characters alone. Therefore, rapid and accurate identification of *Megastigmus* insects and monitoring of their distribution and growth are one of the main measures for the prevention and control of these insects.

DNA barcoding is a method for species identification, based on a relatively short DNA gene fragment with sufficient variation and easy amplification (Folmer et al. 1994, Nuriye et al. 2021). Currently, sequence fragments of mitochondrial DNA (mtDNA: *COI*, *Cytb*, *COII*, *16S* etc.) have been widely used in this method, which can quickly and accurately identify insects (Janzen et al. 2017). In mitochondrial DNA, molecular markers, such as cytochrome c oxidase subunit I (*COI*) and Cytochrome b (*Cytb*), are commonly used to study intra-species, inter-species and even inter-family phylogenetic relationships. They have the characteristics of high evolutionary rate, obvious interspecific variation, but relative conservation within species, good versatility of primers and easy amplification, which can effectively solve some long-standing controversies in morphological taxonomy (Meyer and Wilson 1990, Koch 2010, Cheng et al. 2015, Chen et al. 2020). Therefore, the morphological characteristics combined with two molecular markers (*COI* and *Cytb)* can more effectively solve the problem of classification and identification of *Megastigmus*.

## Materials and methods

The infested cones of *C.chengiana* were collected from Ri’er Township (102°35′ E, 30°59′ N), Xiaojin County, Aba Autonomous Prefecture of Tibetan and Qiang (Ngawa), Sichuan Province, China. The cones were kept in mesh bags, where the spawning adult insects were collected and immediately frozen in liquid nitrogen and preserved at -80°C until DNA extraction. All type specimens are deposited in the Natural History Museum of Sichuan University, Chengdu (NHMSU) with an accession number NHMSU-20201015-XJ.

Terminology of morphological structures in this study mostly follows Roques and Skrzypczynska (2003), Doğanlar and Hassan (2010) and Doğanlar (2015). Terms for surface sculpture follow Steinmann and Zombori (1985). Observations were made using Olympus SZX16 and BX53 microscopes. Photographs of diagnostic characters of the new species were taken by a KEYENCE VHX-5000 digital microscope. Abbreviations of used morphological characters are: POL = posterior ocellus longest diameter, OOL = shortest distance between posterior ocellus and eye margin, dorsal view; OCL = shortest distance between posterior ocellus and occipitial carina, dorsal view; F1–F7 = funicular 1–7; C1–C3 = clava 1–3.

We used Mitoz 3.5.0 software (Meng et al. 2019) to assemble two mitochondrial gene fragments (*COI* and *Cytb*) from genomic sequencing data of *M.daduheensis* sp. n. which was performed with Illumina HiSeq 2000 at Novogene, Beijing, China. Subsequently, a total of 27 *COI* and *Cytb* gene sequences of *Megastigmus* species were found in the National Center for Biotechnology Information (NCBI). Hence, the phylogenetic analysis used genetic data from the above species and one outgroup of *Torymusgeranii* Walker, 1833 (Table 1). The sequences of different species were manually aligned with clustalW in MEGA 7.0 (Kumar et al. 2016). The trimmed alignment was used to estimate the phylogeny by Maximum Likelihood (ML) using IQ-TREE 2.0.4 and an ultrafast bootstrap approximation approach with 1000 replicates (Fu et al. 2014) was used to calculate bootstrap scores for each node (BP) (Minh et al. 2020). MEGA 7.0 software was used to calculate the interspecific genetic distance, based on p-distance and the percentage of base pairs are shared between the species (Kimura 1980, Kumar et al. 2016).

## Taxon treatments

### Megastigmus daduheensis sp. n.

518FB099-DB2E-568D-8EEF-65DD33D10C3D

06D89B26-3CC3-4E8A-979B-8C860627B609

#### Materials

- **Type status:** Holotype. **Occurrence:** occurrenceDetails: CollectionCode: NHMSU-20201015-XJ; GenBank No: *COI* (ON421529) and *Cytb* (ON478368).; individualCount: 1; sex: female; lifeStage: adults; occurrenceID: C3C6985C-AF91-54BA-ACE7-7689077A8ED3; **Taxon:** scientificName: *Megastigmusdaduheensis*; order: Hymenoptera; family: Megastigmidae; genus: Megastigmus; specificEpithet: *daduheensis*; **Location:** country: China; stateProvince: Sichuan; locality: Aba Autonomous Prefecture of Tibetan and Qiang (Ngawa), Xiaojin County, Ri’er Township (102°35′ E, 30°59′ N); locationRemarks: label transliteration:"15 Χ 2020, coll. Wenjing Tao, Wentao Wang and Xue Li"
- **Type status:** Paratype. **Occurrence:** individualCount: 38; sex: female 36 male 2; lifeStage: adults; occurrenceID: E65DFF05-F6EE-579F-9958-FBF8F77337EE; **Taxon:** scientificName: *Megastigmusdaduheensis*; order: Hymenoptera; family: Megastigmidae; genus: Megastigmus; specificEpithet: *daduheensis*; **Location:** country: China; stateProvince: Sichuan; locality: Aba Autonomous Prefecture of Tibetan and Qiang (Ngawa), Xiaojin County, Ri’er Township (102°35′ E, 30°59′ N); locationRemarks: label transliteration:"15 Χ 2020, coll. Wenjing Tao, Wentao Wang and Xue Li"

#### Description

**Figure 1.**
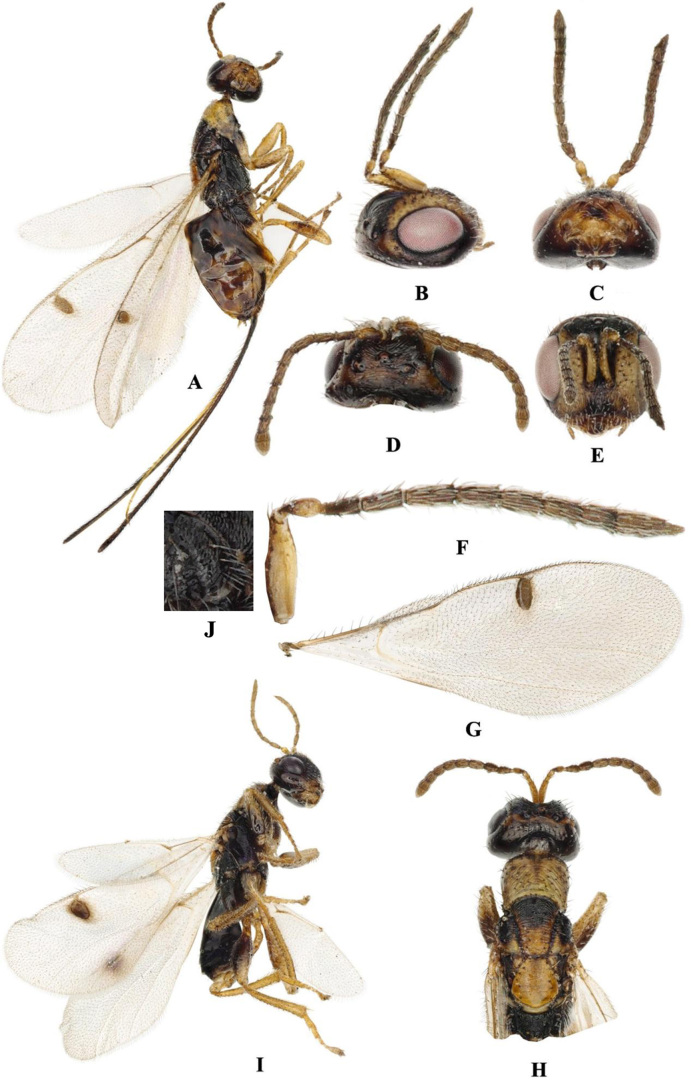
*Megastigmusdaduheensis* sp. n. **A–H** female holotype; **A** Body, lateral view; **B** Head, lateral view; **C** Head, ventral view; **D** Head, dorsal view; **E** Head, frontal view; **F** Antenna; **G** Fore wing; **H** Head and mesosoma, dorsal view; **I** Male, Body, lateral view.

**Female.** Length (body + ovipositor): 2.81 ~ 3.05 + 3.09 ~ 3.17 mm. **Colour**: body (Fig. 1A, H) alternate distribution of yellow, brown and black; head: vertex brown, face yellow, clypeus with apical half dark brown; head antennae light brown; eye reddish-brown; clypeus with apical half dark brown; pronotum brownish-yellow; mesoscutum black anteriorly to brownish-yellow posteriorly; scutellum dark yellow; metanotum light yellow; propodeum black (Fig. 1J); leg brownish-yellow; wing hyaline, vein and the enlarged part of stigmal vein dark brown, stigma light brown; setae of body pale, having black base; hind coxa black, femora and tibia brown; gaster dark brown; ovipositor dark brown.

**Head.** Head about 1.6× as wide as long in dorsal view; whole face with pale, dense setae; frons with many obvious striated sculptures; ocellus arranged in a blunt triangle, POL about twice as long as OOL and OCL; several short setae between ocelli, the rest on other parts of head (Fig. 1B–E). Antennae moniliform, arising from the area below centre of face; scape almost 3.5x as long as width, pedicel about 0.4 of scape length, anellus about 0.25 of pedicel length; relative length/breadth (ratio) of funiculars (F1–F7) as follows: F1 57/29 (1.96), F2 84/38 (2.21), F3 97/39 (2.48), F4 83/46 (1.80), F5 74/47 (1.57), F6 79/45 (1.76), F7 62/45 (1.38); clava (C1–C3) with relative length/breadth (ratio)131/47 (2.79) (Fig. 1F).

**Mesosoma.** Pronotum and mesoscutum with irregular sculpture and orientation of rugae, notauli brown and sparse setae scattered on both sides (Fig. 1H); axillae with oblique longitudinal ridge; scultellum with transverse costula and white setae on both sides; propodeum with oriaceous, callus with long white setae and irregular reticulate sculpture; callus densely covered with white long setae. Basal cell of fore wing with more than ten setae and the lower part of the basal cell closed by a row of setae; marginal vein shorter than postmarginal vein; stigma large and 2.2× as long as width, stigma vein short and 0.11× as long as stigma (Fig. 1G). Hind coxa with white long setae on both sides, femora and tibia densely covered with white setae (Fig. 1A).

**Metasoma.** Gaster not compressed laterally, 1.21 mm long, with sparse bristles scattered with four black horizontal bands and the spacing between the horizontal bands decreasing successively (Fig. 1A). Ovipositor densely covered with brown long setae and 4.5× as long as hind-leg tibia.

**Male.** Body black, 2.4 ~ 2.6 mm long (Fig. 1I). Gaster black, 1.0 mm long, 1.5× as long as hind tibia. Antennae brown. Femora wider than female femora. Marginal vein tan, stigma dark brown. Antennae flagellum about the same length as gaster, funicle length greater than width, anellus about 1/3 of pedicel length. Stigma 1.6× as long as width, stigmal vein short and 0.18× as long as stigma. Other morphology is similar to female.

#### Diagnosis

**Figure 2.**
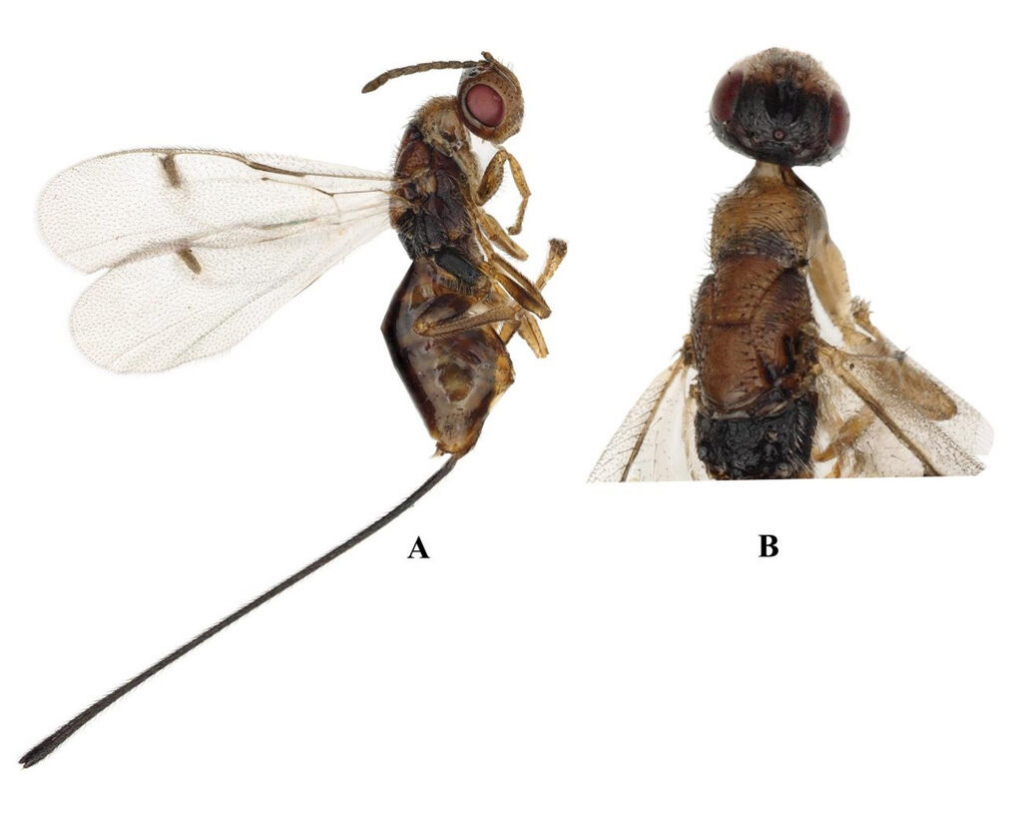
*Megastigmusduclouxianae*, female; **A** Body, lateral view; **B** Head and mesosoma, dorsal view.

**Figure 3.**
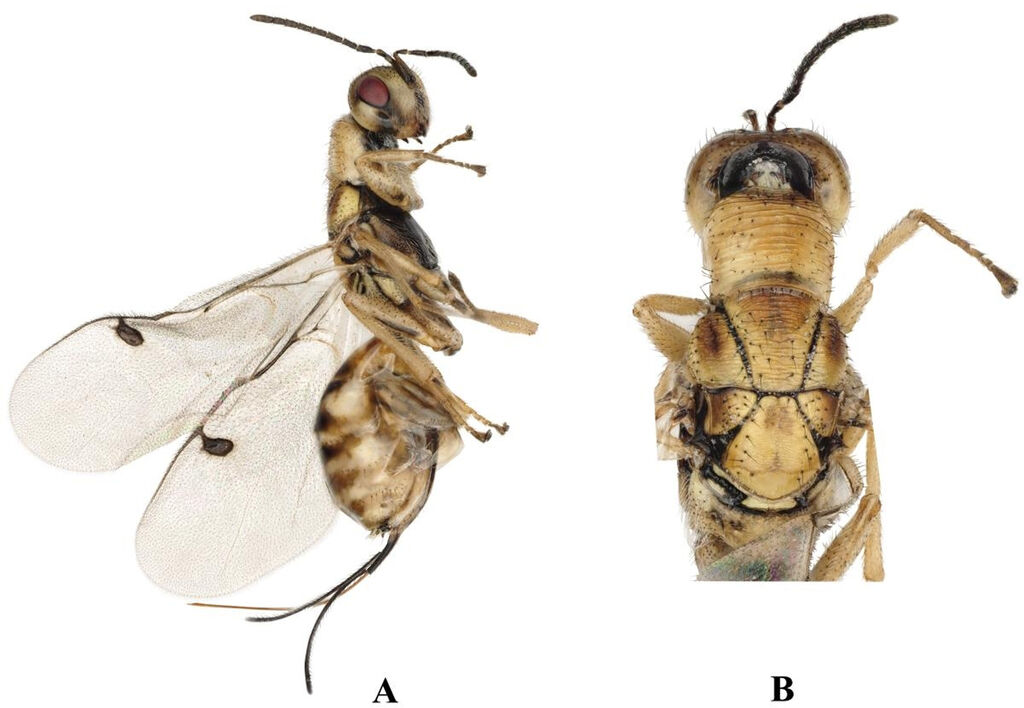
*Megastigmussabinae*, female; **A** Body, lateral view; **B** Head and mesosoma, dorsal view.

The key from China distinguished the females of *Megastigmusdaduheensis* sp. n. by their body length less than 5 mm, ovipositor exceeding body length, stigma neck less than 0.5 times the width of the stigma and scultellum dark yellow. (Fig. 1A, G, E). *Megastigmusdaduheensis* sp. n. is similar to *Megastigmusduclouxianae* Xu & He, 1995 (Fig. 2A, B) and *Megastigmussabinae* Xu & He, 1989 (Fig. 3A,B), but can be distinguished from the latter two species by: 1) stigma light brown, 2.2× as long as width, stigma neck short and 0.11× as long as stigma; 2) ovipositor 4.5× as long as metatibia; and 3) mesosctum black anteriorly to brownish-yellow posteriorly.

#### Etymology

The new species is named after its type locality, Daduhe, Sichuan Province.

#### Biology

The eggs are laid in the cones of *Cupressuschengiana* S.Y. Hu, feeding on the nuts and the adults fly out of the cones from October to November every year.

## Identification Keys

### Key to species of *Megastigmus* from China (females)

**Table:** 

1	Body length greater than 5 mm	*M.sinensis* Sheng, 1989
–	Body length less than 5 mm	2
2	Ovipositor equals or exceeds body length	3
–	Ovipositor shorter than body length	8
3	Stigma neck more than 0.5 times the width of the stigma	*M.aculeatus* Swederus, 1975
–	Stigma neck less than 0.5 times the width of the stigma	4
4	Marginal vein shorter than postmarginal vein length	5
–	Marginal vein equals or exceeds postmarginal vein length	6
5	Scultellum dark brown	*M.duclouxianae* Xu & He, 1995
–	Scultellum dark yellow	*M.daduheensis* sp. n.
6	Width of head about 1.17 times the length in the ventral view	*M.carinus* Xu & He, 1995
–	Head about twice as wide as long in the ventral view	7
7	Pronotum with two yellow spots	*M.likiangensis* Roques & Sun, 1995
–	Pronotum without spots	*M.* sp. (Host:*Tsugaforresttii*)
8	Mid-lobe of mesoscutum with short longitudinal ridge	*M.ezomatsuanus* Hussey & Kamijo, 1958
–	Mid-lobe of mesoscutum with transverse costulae	9
9	Ovipositor longer than gaster length	*M.cellus* Xu & He, 1995
–	Ovipositor equal to or shorter than gaster length	10
10	Scultellum black	11
–	Scultellum yellow or brown	13
11	Pedicel of antennae longer than F1 length	*M.lasiocarpae* Crosby, 1913
–	Pedicel of antennae equal to or shorter than F1 length	12
12	Marginal vein equal to postmarginal vein length	*M.* sp. (Host: *Sabina recurva var.coxii*)
–	Marginal vein shorter than postmarginal vein length	*M.pictus* Foerster, 1841
13	Stigma neck length equal to the width of the stigma	*M.pseuclotsugaphilus* Xu & He, 1995
–	Stigma neck length shorter than the width of the stigma	14
14	Body orange yellow	15
–	Body yellowish-brown or light yellow	16
15	Head with dark spots	*M.sabinae* Xu & He, 1989
–	Head without dark spots	*M.formosana* Roques & Pan, 2005
16	Transscutal articulation of scutellum with irregular reticulate sculpture	*M.pseuclomali* Xu & He, 1995
–	Transscutal articulation of scutelluma with striped or smooth sculpture	17
17	F7 more than twice as long as wide	*M.pingii* Roques & Sun, 1995
–	F7 less than twice as long as wide	18
18	Pronotum 1.9 times longer than width	*M.rigidae* Xu & He, 1998
–	Pronotum 1.1-1.3 times longer than width	*M.cryptomeriae* Yano, 1918

## Analysis

### Phylogenetic relationship

**Figure 4.**
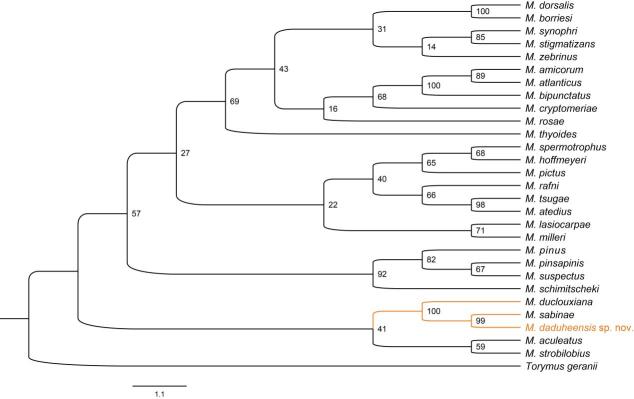
Phylogenetic trees of *Megastigmus* inferred by Maximum Likelihood (ML) methods, based on mitochondrial data (*COI* + *Cytb*).

*Megastigmus* is sometimes difficult to classify accurately because of its small size and similar morphological characteristics. Therefore, the phylogenetic relationships are important to resolve species boundaries. Here, the existing mitochondrial data (*COI* + *Cytb*) was used to construct a molecular phylogenetic tree of *Megastigmus*, so as to verify the taxonomic status of the new species described above (Fig. 4). In terms of phylogenetic relationships, *M.daduheensis* sp. nov. and *M.sabinae* were sister species and then they constituted one clade with *M.duclouxiana*. This result is consistent with morphological identification.

### Genetic distance

In recent years, genetic distance has been regarded as one of the main characteristics of Hymenoptera species classification (Takahashi et al. 2018, Kitnya et al. 2022). The criteria proposed by Hebert et al. (2003) showed that the intraspecific genetic distance of invertebrate was less than 0.02 and the interspecific genetic distance was more than 10 times the intraspecific genetic distance (0.2). Thus, MEGA 7.0 was used to calculate pairwise genetic distances (Table 2) and common base pairs between species for 28 species of the *Megastigmus*, based on *COI* and *Cytb*. The results showed that the interspecific genetic distance base on the *COI* and *Cytb* were 0.01 ~ 0.60 and 0.02 ~ 0.59, with an average of 0.163 and 0.132, respectively. The genetic distances (*COI*/*Cytb*) of *M.daduheensis* sp. nov. with *M.duclouxiana* and *M.sabinae* were 0.56/0.55 and 0.54/0.57 and the percentages of shared base pairs (*COI*+*Cytb*) were 76.3% and 76.8%, respectively. Molecular evidence confirmed *M.daduheensis* sp. nov. was distantly related to *M.duclouxiana* and *M.sabinae* and it was a separate species.

## Discussion

The identification of species based on morphological differences is essential to insect taxonomy. Nevertheless, the traditional classification method has drawbacks when it comes to tiny and similar-looking species, while molecular data can make up for the shortcomings of this method. Currently, many studies have stated explicitly the appropriateness of mtDNA in resolving the relationships amongst subspecies or closely-related species (Sperling and Hickey 1995, Davison et al. 2001, Wahlberg et al. 2003). For instance, *Megastigmusspermotrophusnigrodorsatus* has been considered until now as a subspecies of *M.spermotrophus*, but molecular results (mean distances of 3.3% in *Cytb* and 0.3% in *28S*) suggested that the species actually could be a distinct species rather than a subspecies of *M.spermotrophus* (Auger-Rozenberg et al. 2010). Similarly, two specimens from Greece and Kenya were identified as *Megastigmuspistaciae*, but Roques et al. (2017) study showed that they diverged by 4.5% in the *COI* data and the two are probably sibling species. Therefore, the combination of the two methods can make insect identification more accurate, which is of great significance for improving the efficiency and accuracy of species identification.

In this paper, morphological characters and molecular data (*COI* and *Cytb*) were used to classify parasitic wasps from *Cupressuschengiana* S.Y. Hu. Both methods confirm that *M.daduheensis* sp. nov. is a separate species, which is clearly different from other species in the genus and has a certain degree of diversification. Although *M.daduheensis* sp. n. was similar to *M.sobinae* and *M.duclouxiana* in morphology, it can be distinguished from other species, based on characters on the mesoscutum, stigma and ovipositor. *M.daduheensis* sp. nov. and *M.sabinae* were sister species, based on the phylogenetic relationship and genetic distance and, together, they constituted one clade with *M.duclouxiana*. This result is consistent with previous studies and supports *Megastigmus* on Cupressaceae plants formed a distinct clade (Auger-Rozenberg et al. 2010). Roques et al. (2017) has calculated intra-group and inter-group distances of *Megastigmus*, based on the *COI* dataset and the lower values were observed within groups rather than between groups, with values ranging from 6% to 7.9%. However, in our study, the average pairwise genetic distance (*COI*/*Cytb*: 0.16/0.13) of *Megastigmus* species was higher and the genetic distance of *M.daduheensis* sp. nov. supported it as a separate species.

This study not only increased the species diversity of *Megastigmus*, but also provided the genetic information of *M.daduheensis* sp. nov. and enriched the genetic database. However, our study only obtained *COI* and *Cytb* genes of this genus, which contained incomplete genetic information. Therefore, it can be combined with other mitochondrial or nuclear genes for a comprehensive analysis and verification in the future. In addition, there are a large number of species within this genus, but most of them are studied, based on morphology, ecology and physiology, while molecular data is very scarce. Hopefully, we will be able to collect more samples and obtain molecular data to make up for the shortfall.

## Supplementary Material

XML Treatment for Megastigmus daduheensis

## Figures and Tables

**Table 1.** List of the mitochondrial genes analysed in the present study.

**Family/ Genus**	**Species**	**GenBank No. (*COI*)**	**GenBank No. (*Cytb*)**
*Megastigmus*	*M.daduheensis* sp. n.	ON421529	ON478368
	*M.aculeatus*	KF531842.1	JQ756668.1
	*M.amicorum*	KF531835.1	AY898663.1
	*M.atedius*	KF531848.1	AY898691.1
	*M.atlanticus*	KF531836.1	AY898669.1
	*M.bipunctatus*	KF531837.1	AY898664.1
	*M.borriesi*	KF531852.1	AY898681.1
	*M.cryptomeriae*	KF531841.1	AY898699.1
	*M.dorsalis*	AY317240.1	KX980153.1
	*M.duclouxiana*	ON421527	ON478366
	*M.hoffmeyeri*	KF531845.1	AY898695.1
	*M.lasiocarpae*	KF531853.1	AY898671.1
	*M.milleri*	KJ535739.1	AY898672.1
	*M.pictus*	KF531847.1	AY898696.1
	*M.pinsapinis*	KF531855.1	AY898684.1
	*M.pinus*	KF531850.1	AY898673.1
	*M.rafni*	KF531846.1	AY898678.1
	*M.rosae*	KF531840.1	AY898701.1
	*M.sabinae*	ON421528	ON478367
	*M.schimitscheki*	KF531854.1	AY898686.1
	*M.spermotrophus*	KF531844.1	AY898694.1
	*M.stigmatizans*	JQ417143.1	AY898703.1
	*M.strobilobius*	MN122037.1	AY898697.1
	*M.suspectus*	KF531856.1	AY898688.1
	*M.synophri*	FJ026673.1	FJ026729.1
	*M.thyoides*	KF531851.1	AY898667.1
	*M.tsugae*	KF531849.1	AY898682.1
	*M.zebrinus*	MN165915.1	KC710378.1
Torymidae	*Torymusgeranii*	HM574310.1	GU123595.1

**Table 2.** Pairwise genetic distances of *COI* and *Cytb* amongst 28 species of *Megastigmus*.

**(*COI*/ *Cytb*)**	**1**	**2**	**3**	**4**	**5**	**6**	**7**	**8**	**9**	**10**
2	0.09/0.11									
3	0.56/0.55	0.54/0.57								
4	0.60/0.17	0.59/0.14	0.12/0.56							
5	0.56/0.11	0.54/0.09	0.10/0.57	0.12/0.17						
6	0.57/0.05	0.56/0.10	0.10/0.55	0.13/0.17	0.10/0.10					
7	0.55/0.05	0.53/0.09	0.10/0.54	0.14/0.16	0.10/0.09	0.06/0.04				
8	0.56/0.10	0.54/0.08	0.11/0.56	0.13/0.14	0.10/0.09	0.12/0.10	0.12/0.08			
9	0.56/0.11	0.55/0.07	0.10/0.56	0.12/0.15	0.11/0.09	0.11/0.10	0.12/0.10	0.10/0.06		
10	0.56/0.10	0.55/0.07	0.11/0.55	0.11/0.14	0.09/0.07	0.11/0.09	0.11/0.08	0.08/0.04	0.06/0.04	
11	0.58/0.12	0.57/0.14	0.09/0.56	0.11/0.14	0.11/0.13	0.10/0.12	0.10/0.12	0.10/0.13	0.10/0.12	0.08/0.12
12	0.55/0.11	0.54/0.10	0.10/0.55	0.11/0.17	0.07/0.08	0.10/0.10	0.10/0.10	0.06/0.06	0.07/0.07	0.06/0.05
13	0.59/0.11	0.58/0.09	0.22/0.57	0.25/0.17	0.21/0.09	0.23/0.10	0.22/0.09	0.23/0.07	0.25/0.08	0.22/0.06
14	0.56/0.15	0.54/0.12	0.12/0.58	0.11/0.17	0.11/0.15	0.12/0.14	0.12/0.14	0.12/0.15	0.14/0.14	0.12/0.14
15	0.57/0.15	0.55/0.13	0.10/0.57	0.12/0.13	0.10/0.17	0.13/0.17	0.13/0.16	0.10/0.15	0.12/0.14	0.10/0.15
16	0.57/0.10	0.54/0.09	0.10/0.56	0.11/0.16	0.09/0.09	0.11/0.11	0.12/0.10	0.07/0.07	0.06/0.07	0.06/0.05
17	0.56/0.10	0.54/0.08	0.09/0.56	0.11/0.15	0.09/0.08	0.12/0.09	0.10/0.09	0.07/0.05	0.07/0.05	0.02/0.04
18	0.57/0.11	0.55/0.09	0.10/0.56	0.12/0.17	0.09/0.09	0.11/0.11	0.12/0.10	0.07/0.07	0.07/0.07	0.06/0.05
19	0.57/0.10	0.55/0.09	0.11/0.56	0.12/0.16	0.09/0.09	0.12/0.10	0.12/0.09	0.09/0.06	0.08/0.07	0.06/0.05
20	0.56/0.10	0.55/0.08	0.10/0.55	0.10/0.14	0.09/0.08	0.10/0.08	0.12/0.08	0.08/0.05	0.07/0.05	0.07/0.04
21	0.56/0.12	0.54/0.11	0.09/0.56	0.10/0.17	0.09/0.11	0.10/0.10	0.09/0.10	0.09/0.10	0.10/0.08	0.08/0.09
22	0.57/0.10	0.54/0.09	0.09/0.55	0.12/0.13	0.08/0.10	0.11/0.09	0.11/0.08	0.07/0.06	0.09/0.06	0.07/0.05
23	0.58/0.07	0.56/0.11	0.12/0.56	0.14/0.20	0.11/0.11	0.07/0.06	0.06/0.06	0.13/0.09	0.12/0.11	0.10/0.09
24	0.55/0.10	0.53/0.07	0.08/0.55	0.11/0.14	0.09/0.08	0.10/0.08	0.10/0.07	0.07/0.04	0.07/0.03	0.06/0.03
25	0.56/0.13	0.54/0.12	0.10/0.57	0.11/0.17	0.12/0.13	0.11/0.11	0.13/0.12	0.09/0.11	0.10/0.11	0.09/0.11
26	0.56/0.12	0.54/0.10	0.10/0.55	0.10/0.16	0.09/0.11	0.12/0.11	0.13/0.09	0.06/0.06	0.07/0.07	0.07/0.05
27	0.56/0.09	0.54/0.07	0.09/0.56	0.10/0.14	0.09/0.09	0.11/0.09	0.11/0.08	0.05/0.03	0.07/0.05	0.06/0.04
28	0.56/0.11	0.54/0.08	0.09/0.56	0.11/0.15	0.07/0.08	0.09/0.09	0.09/0.08	0.07/0.05	0.07/0.06	0.05/0.04
**(*COI*/ *Cytb*)**	**11**	**12**	**13**	**14**	**15**	**16**	**17**	**18**	**19**	**20**
12	0.10/0.13									
13	0.25/0.13	0.21/0.06								
14	0.11/0.15	0.10/0.16	0.24/0.15							
15	0.10/0.15	0.09/0.15	0.21/0.18	0.09/0.13						
16	0.10/0.13	0.03/0.04	0.22/0.07	0.12/0.15	0.090.15					
17	0.08/0.12	0.05/0.06	0.22/0.07	0.11/0.15	0.10/0.15	0.06/0.07				
18	0.10/0.13	0.03/0.04	0.22/0.07	0.12/0.15	0.09/0.14	0.01/0.02	0.06/0.07			
19	0.11/0.13	0.06/0.07	0.24/0.09	0.11/0.15	0.10/0.17	0.07/0.08	0.05/0.06	0.07/0.07		
20	0.09/0.12	0.05/0.06	0.23/0.07	0.1/0.15	0.11/0.16	0.06/0.07	0.06/0.05	0.06/0.08	0.07/0.06	
21	0.08/0.14	0.07/0.11	0.23/0.12	0.09/0.15	0.09/0.17	0.09/0.10	0.08/0.09	0.09/0.10	0.09/0.10	0.08/0.10
22	0.09/0.11	0.06/0.08	0.22/0.09	0.11/0.10	0.09/0.12	0.07/0.08	0.07/0.07	0.07/0.07	0.07/0.07	0.07/0.06
23	0.11/0.13	0.11/0.10	0.24/0.11	0.13/0.16	0.12/0.18	0.12/0.11	0.10/0.11	0.13/0.11	0.12/0.10	0.12/0.10
24	0.09/0.11	0.05/0.05	0.23/0.05	0.12/0.14	0.10/0.14	0.06/0.06	0.05/0.04	0.06/0.06	0.08/0.05	0.05/0.03
25	0.09/0.13	0.09/0.12	0.23/0.11	0.13/0.14	0.10/0.14	0.09/0.12	0.09/0.11	0.09/0.12	0.10/0.13	0.09/0.11
26	0.10/0.15	0.05/0.07	0.23/0.08	0.10/0.17	0.09/0.18	0.06/0.08	0.06/0.07	0.06/0.07	0.07/0.07	0.06/0.06
27	0.09/0.13	0.04/0.06	0.23/0.06	0.11/0.15	0.09/0.16	0.05/0.07	0.05/0.05	0.06/0.07	0.06/0.05	0.06/0.05
28	0.09/0.12	0.04/0.06	0.23/0.07	0.09/0.15	0.10/0.15	0.06/0.07	0.04/0.05	0.06/0.07	0.06/0.05	0.06/0.04
**(*COI*/ *Cytb*)**	**21**	**22**	**23**	**24**	**25**	**26**	**27**			
22	0.07/0.09									
23	0.10/0.11	0.11/0.10								
24	0.07/0.08	0.07/0.05	0.11/0.09							
25	0.09/0.11	0.10/0.10	0.12/0.13	0.10/0.10						
26	0.08/0.11	0.07/0.08	0.12/0.12	0.06/0.05	0.08/0.12					
27	0.08/0.10	0.07/0.07	0.11/0.09	0.06/0.03	0.08/0.11	0.04/0.06				
28	0.06/0.09	0.05/0.06	0.10/0.10	0.06/0.03	0.08/0.10	0.06/0.05	0.05/0.05			

Note: *COI* fragment before backslash (/) and *Cytb* fragment after backslash. 1 *M.Sabinae*; 2 *M.duclouxiana*; 3 *M.daduheensis* sp. nov.; 4 *M.dorsalis*; 5 *M.aculeatus*; 6 *M.amicorum*; 7 *M.bipunctatus*; 8 *M.pinus*; 9 *M.spermotrophus*; 10 *M.tsugae*; 11 *M.zebrinu*; 12 *M.schimitscheki*; 13 *M.strobilobius*; 14 *M.synophri*; 15 *M.stigmatizans*; 16 *M.pinsapinis*; 17 *M.atedius*; 18 *M.suspectus*; 19 *M.rafni*; 20 *M.pictus*; 21 *M.cryptomeriae*; 22 *M.thyoides*; 23 *M.atlanticus*; 24 *M.hoffmeyeri*; 25 *M.rosae*; 26 *M.lasiocarpae*; 27 *M.milleri*; 28 *M.borriesi*.
